# A comparison of long-term results after Baerveldt 250 implantation in advanced uveitic vs. other forms of glaucoma

**DOI:** 10.1007/s00417-022-05612-x

**Published:** 2022-03-07

**Authors:** Ioana Maria Cazana, Daniel Böhringer, Thomas Reinhard, Alexandra Anton, Thomas Ness, Jan Lübke

**Affiliations:** 1 Department of Ophthalmology, University Hospital of Zurich, Frauenklinikstrasse 24, 8091 Zurich, Switzerland; 2 Eye Center, Medical Center, University of Freiburg, Faculty of Medicine, Freiburg, Germany; 3 ADMEDICO Eye Center, Olten, Switzerland

**Keywords:** Baerveldt implant, Secondary inflammatory glaucoma, Uveitic glaucoma, Shunts, Drainage devices

## Abstract

**Introduction:**

Uveitic glaucoma remains challenging despite medical and surgical advancements and can potentially lead to blindness if left uncontrolled. Conservative alternatives as well as microinvasive surgeries can postpone the necessity of a highly invasive intervention. However, such procedures are still necessary to treat some refractive glaucoma cases. Since previous studies have reported excellent results following the primary implantation of glaucoma drainage devices, it was our study’s aim to evaluate long-term results following a Baerveldt 250 implantation in highly complex and surgically burdened uveitic glaucoma eyes (UG) and compare these to a similar population suffering from other forms of glaucoma (OFG).

**Material and methods:**

We performed a retrospective analysis of all eyes (UG vs. OFG) following a Baerveldt 250 implant between 2013 and 2019. Efficacy parameters as well as post-operative complication data were extracted from our electronic data system for statistical analysis.

**Results:**

A total of 62 eyes were included in our study (24 UG and 38 OFG). UG baseline mean IOP was 35.04 mmHg (± 11.85 mmHg) with 3.08 (± 1.13) topical agents, and OFG was 32.63 mmHg (± 7.74 mmHg) with 2.68 (± 1.28) topical agents. A majority of eyes also required systemic acetazolamide (UG: 79% OFG: 87%) and had undergone at least one glaucoma-related operation prior to the Baerveldt 250 implant ((UG: 1.21 (± 0.66)), OFG: 1.74 (± 1.33)). At the median follow-up period (UG 592, OFG 764 days), 52.5%/32.5% of UG/OFG cases showed qualified success (IOP below 21 mmHg with either topical or/and systemic medication), 15%/30% no longer required topical medication, and 47.5% /47.5% were free of acetazolamide systemically. Moreover, 75%/72.5% of eyes experienced no further pressure-related surgical event. Although sight-threatening complications such as corneal and macular edema were reported in both groups, most either maintained or improved their visual acuity at the last follow-up (58.33%/57.89%).

**Conclusion:**

The Baerveldt 250 implant is shown to be both effective and safe for advanced glaucoma cases in uveitis and other forms. No further glaucoma-related surgery is required in the majority of eyes in either group within a follow-up period of almost 2 years. Despite sight-threatening complications such as macular and corneal edema, visual acuity can be either maintained or improved in most eyes.


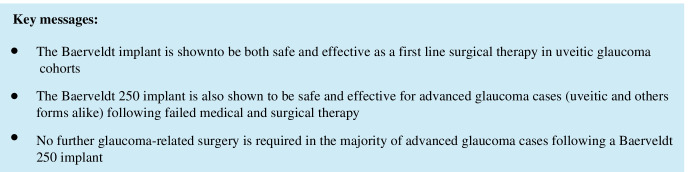


## Introduction

With a 10–20% prevalence, glaucoma is a common and severe complication of uveitis [1, 2]. Despite ongoing advancements, its management remains a complex challenge, which requires addressing both the underlying condition and an elevated intraocular pressure (IOP) simultaneously. A reduced outflow capacity after recurrent inflammation coupled by a hypertensive response to corticosteroids is responsible for the persistence of an elevated IOP despite intensive medical therapy [3–6]. Furthermore, surgical interventions are necessary in up to 35% of adults and 59% of children due to medical treatment failure [7].

A myriad of surgical options with comparable efficacies are currently available [8–14]. However, chronic inflammation can compromise the long-term outcome following both trabeculectomy ab externo and interno, and cycloablative techniques remain a last resort because of their irreversible nature [8–14]. Since a poorly controlled glaucoma may lead to blindness, urgent and aggressive forms of treatment are necessary. Glaucoma drainage devices (GDD) offer an efficient and safe alternative for combating refractory hypertension due to uveitis [9]. Specifically, the Baerveldt implant (BI) is shown to be of significant relevance in resolving such cases [15–17]. Long-term performance reports are relatively scarce. Additionally, to our knowledge, none includes highly diseased and surgically burdened study populations following a small Baerverldt 250 implant (BI-250). Filling this gap, we evaluate the long-term efficiency and safety following BI-250 in complex eyes suffering from glaucoma due to uveitis (UG) vs. other forms (OFG).

## Material and methods

### Retrospective data

This single-center retrospective study was performed at the Eye Center of the Medical Center, University of Freiburg. Each tenant of the Declaration of Helsinki was upheld. Following ethics committee approval (vote no. 619/19), data was acquired based on our electronic patient management system. All patients that underwent a BI-250 between 2013 and 2019 were identified. Their last pre-operative and all follow-up medical records were reviewed, and relevant information was entered in our database. From the last pre-operative examination, age, gender, visual acuity, type of uveitis, IOP, use of medication (topical and systemic), pre-BI-250 surgeries, lens status, and presence of macular and corneal edema were extracted. At follow-up, data included as follows: visual acuity, IOP, use of medication (topical and systemic), post-BI-250 surgeries, presence of macular and corneal edema/transplantation rates, and surgical complications.

Our hospital routinely uses Goldmann applanation tonometry (Haag-Streit, Köniz, Switzerland) to measure IOP in glaucoma patients. The device is calibrated according to the manufacturer’s recommendations. A complete success was defined as an IOP below 21 mmHg without either topical or systemic medication, while a qualified success was defined as an IOP below 21 mmHg with either topical and/or systemic medication. Hypotony was considered as an IOP below 5 mmHg. Post-operative IOP spikes are common in the first month due to tube occlusion. Therefore, IOP was reported starting in the second post-operative month.

The total number of IOP lowering agents resulted from the sum of each category of topical drugs (beta-blockers, prostaglandin-analogues, carbonic anhydrase inhibitors, alpha2-agonists). Agents present in fixed combination eye drops were regarded individually and oral acetazolamide was considered separately.

Cases showing ophthalmoscopically ambiguous macular findings despite a stable visual acuity and those with an unexplained worsened visual acuity received further testing via optical coherence tomography (OCT) using a Spectralis OCT (Heidelberg Engineering, Dossenheim, Germany). Treatment proceeded according to cause. The presence of corneal edema drastically impacts post-operative visual outcomes and was therefore evaluated through slit lamp microscopy and endothelial cell count. Furthermore, post-BI-250 corneal transplantations were tabulated for native and grafted eyes of both groups. Lastly, regardless of cause, we considered any visual acuity change of 0.2 logMAR (2 lines) to be significant.

### Surgical procedure

A total of four surgeons performed all Baerveldt implantations following the same surgical procedure. After an initial limbus-based conjunctival incision in the inferonasal quadrant, the BI, BG103-250 (62) (Abbott Medical Optics Inc., Santa Ana, CA, USA) was secured with its wings beneath the inferior and medial rectus muscles and its anterior edge 11 mm posterior to the limbus, using two dafilon 6–0 sutures. Patency was ensured through irrigation, and tube occlusion was performed using a vicryl rapide 6–0 suture in order to prevent post-operative hypotony. After the tube was individually shortened in order for it to lay comfortably in the anterior chamber avoiding pupil-obstruction, a scleral flap was prepared and the tube was then inserted in the anterior chamber using a 23-gauge needle for tracking. The tube was attached to the globe using nylon 10–0 sutures and the scleral flap was secured and conjunctiva closed using vicryl 7–0 sutures. At the conclusion of the procedure, an intracameral steroid injection was administered via a paracentesis.

### Statistical analysis

All statistical analyses were performed using R statistical software [18]. Differences in patient’s baseline characteristics were analyzed using either the independent *t* test for continuous variables or the chi-square test for categorical variables. *P* values below 0.05 were considered statistically significant. Next, Kaplan–Meier curves were employed to provide a visualization of post-operative outcomes between both groups, and the results at the median follow-up time were extrapolated (UG vs. OFG).

## Results

### Baseline characteristics

A total of 62 eyes were included in our study (24 UG and 38 OFG). Of the 24 UG eyes, 54% presented with anterior-, 29% with intermediate-, and 17% with pan-uveitis. Both groups comprised of relatively young patients, with a higher female representation in the UG group. The mean IOP was 35.04 mmHg (± 11.85 mmHg) with 3.08 (± 1.13) topical agents in the UG group, 32.63 mmHg (± 7.74 mmHg) with 2.68 (± 1.28) in the OFG group. 79%/87% of eyes respectively followed a systemic acetazolamide regimen before the operation. On average, patients underwent 1 to 2 glaucoma operations before receiving a BI-250 ((UG: 1.21 (± 0.66)), OFG: 1.74 (± 1.33)). Further baseline characteristics can be found in Table 1.

**Table 1:** Baseline pre-operative characteristics of the study population

Baseline characteristics			
	UG	OFG	*p*-values
Number	24	38	
Sex (%)			0.08
Female Male	62.5 37.5	39.5 60.5	
Age (years)	52.79	51.11	0.93
Lens status			0.51
Phakic Pseudophakic Aphakic	3 19 2	2 34 2	
Keratoplasty total (%) Penetrating keratoplasty DMEK DSAEK	8.33 1 1 0	42.10 13 2 1	0.03
Macular edema (%)	12.50	2.63	0.31
Uveitis location (%)			
Anterior Intermediate Pan-uveitis	54 29 17		
IOP (mmHg)	35.04 (± 11.85)	32.63 (± 7.74)	0.58
Topical agents	3.08 (± 1.13)	2.68 (± 1.28)	0.25
Systemic acetazolamide (%)	79%	87%	0.42
Visual acuity (log MAR)	0.38 (± 0.42)	1.12 (± 0.83)	< 0.001
Glaucoma operations before BI 250 Traculectomy ab interno Trabeuculectomy ab externo Cyclophotocoagulation/cryocoagulation Iridectomy Molteno implant Cypass implant Goniotomy XEN implant SLT	1.21 (± 0.66) 19 7 0 0 0 0 0 0 1	1.74 (± 1.33) 14 14 15 4 1 2 1 1 0	0.07
Median follow-up (days)	592	764	0.10
Mean uveitis duration to surgery (months)	146.72		
Other form of glaucoma (%):			
Congenital glaucoma Developmental glaucoma Iridocorneal endothelial syndrome Neovascular glaucoma Primary open angle glaucoma Angle closure glaucoma Pseudoexfoliative glaucoma Post-traumatic glaucoma Post-operative glaucoma		8 3 8 3 26 8 5 13 26	

### Post-operative results

**Fig. 1:**
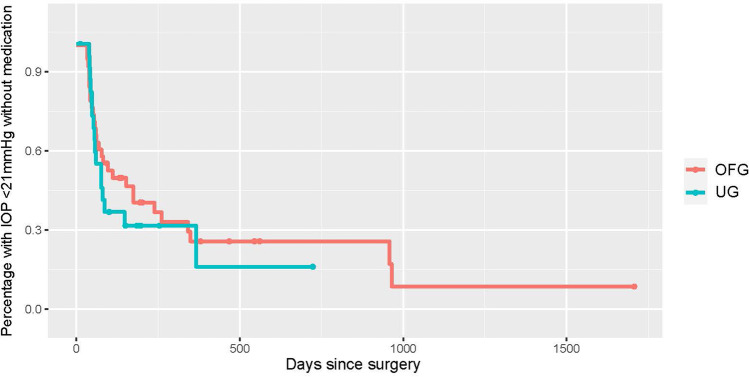
Kaplan–Meier curve displaying the complete success rate for the UG group (teal) vs. OFG group (red) (IOP < 21 mmHg without topical medication or systemic acetazolamide). Censored cases appear as a dot

**Fig. 2:**
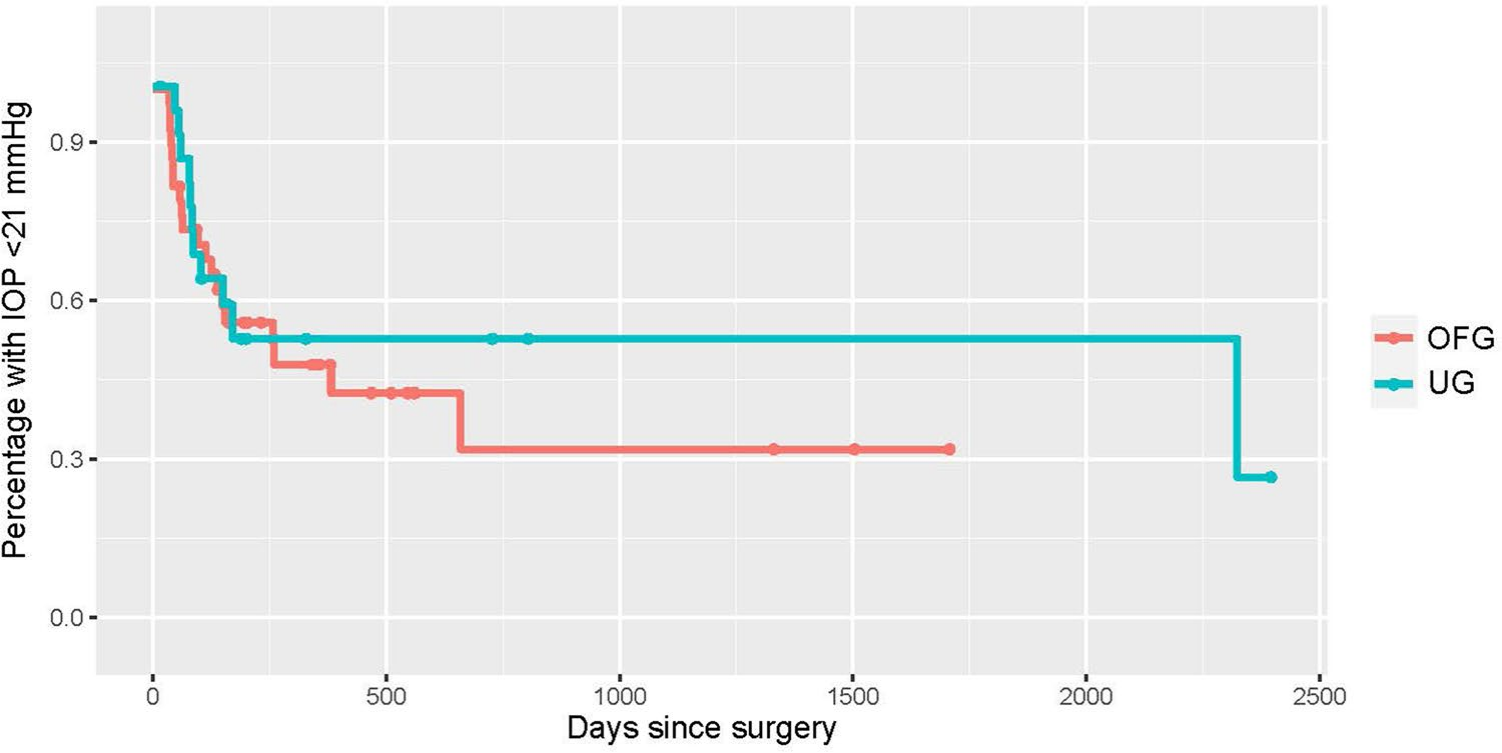
Kaplan–Meier curve displaying the qualified success rate for the UG group (teal) vs. OFG group (red) (IOP < 21 mmHg with either topical medication and/or systemic acetazolamide). Censored cases appear as a dot

**Fig. 3:**
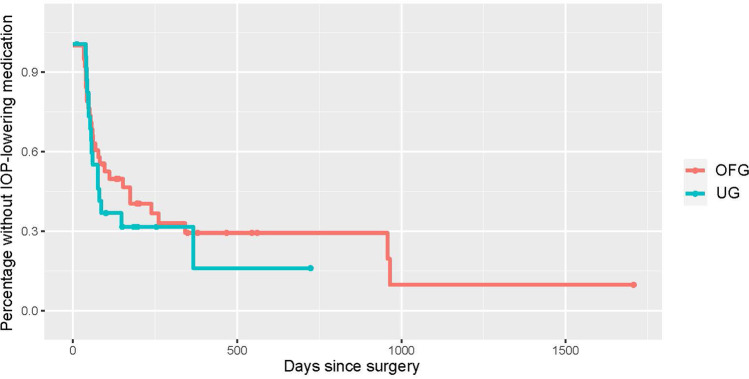
Kaplan–Meier curve depicting cases post-BI-250 without topical IOP lowering medication for the UG group (teal) vs. OFG group (red). Censored cases appear as a dot

**Fig. 4:**
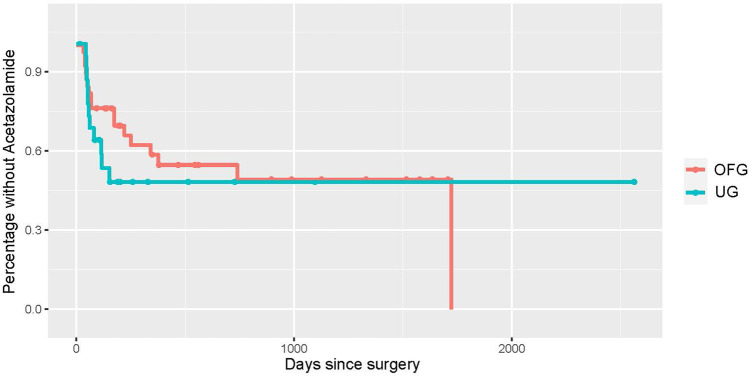
Kaplan–Meier curve depicting cases post-BI-250 without systemic acetazolamide for the UG group (teal) vs. OFG group (red). Censored cases appear as a dot

**Fig. 5:**
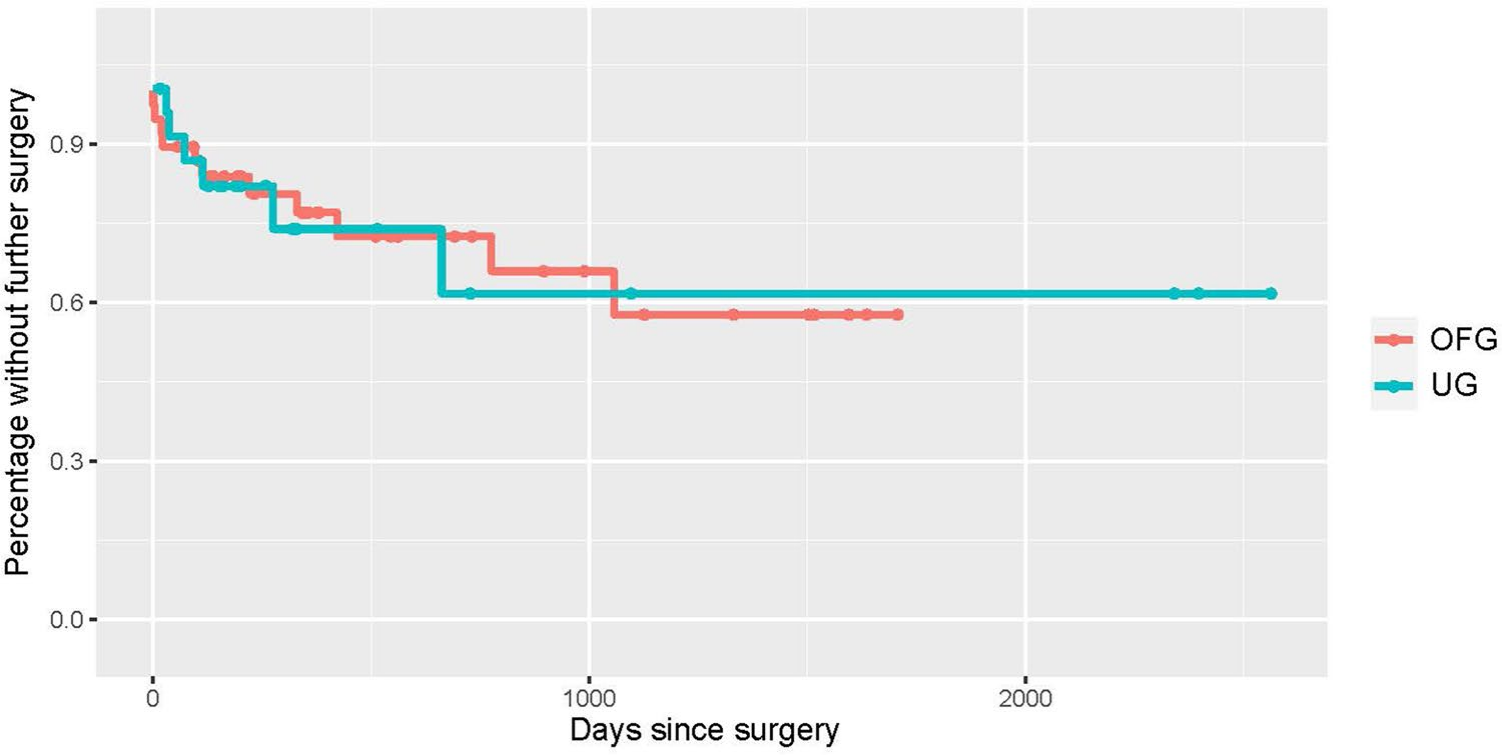
Kaplan–Meier curve depicting cases post-BI-250 without further glaucoma-related surgeries for the UG group (teal) vs. OFG group (red). Censored cases appear as a dot

The median follow-up period was 592/764 days for patients in the UG/OFG cohort. At this time, 15%/25% registered a complete success, and 52.5%/32.5% a qualified success. 15% of the UG and 30% of the OFG eyes no longer required topical medication, and 47.5% in both were free of acetazolamide systemically. 75%/72.5% of eyes experienced no further pressure-related surgical event. Almost all cases requiring further surgery underwent cyclophotocoagulation. Kaplan–Meier curves were generated for each of the aforementioned efficacy criteria (see Figs. 1, 2, 3, 4, and 5).

**Fig. 6:**
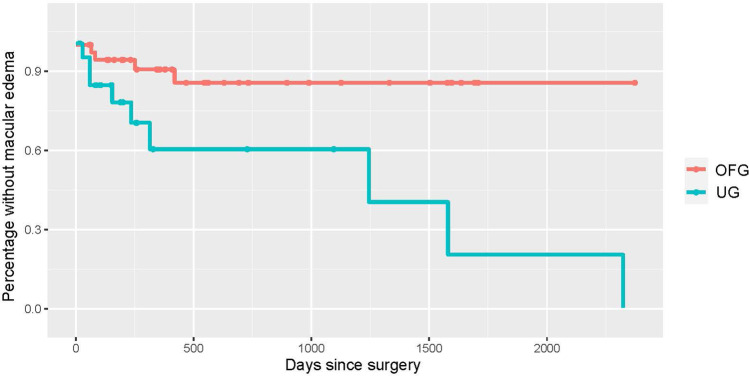
Kaplan–Meier curve illustrating macular edema prevalence post-BI-250 for the UG group (teal) vs. OFG group (red). Censored cases appear as a dot

**Fig. 7:**
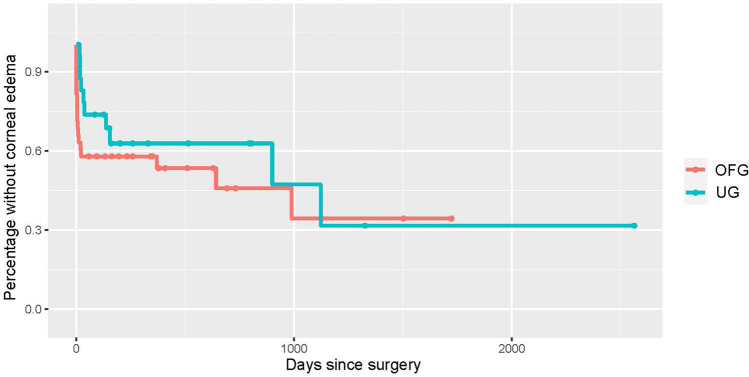
Kaplan–Meier curve depicting corneal edema prevalence post-BI-250 for the UG group (teal) vs. OFG group (red). Censored cases appear as a dot

**Fig. 8:**
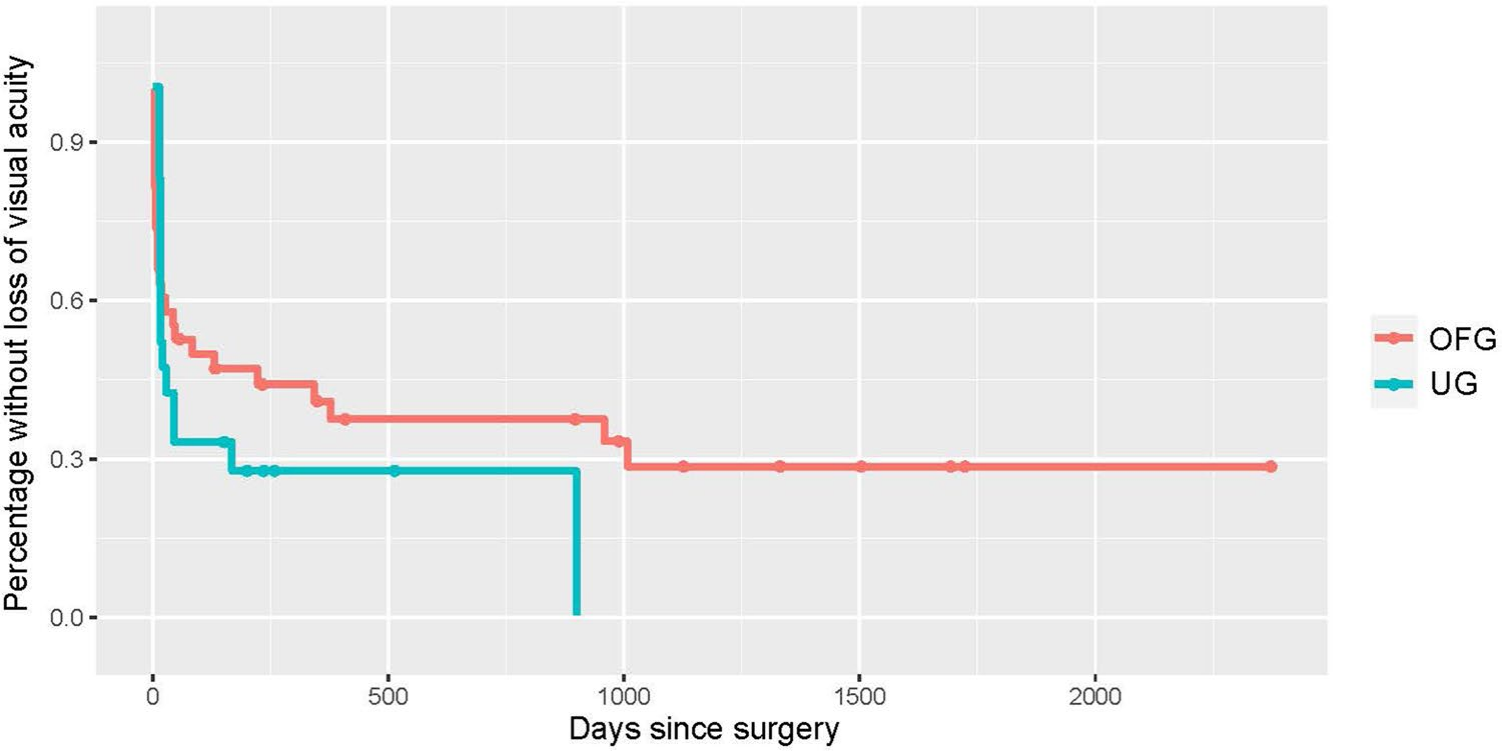
Kaplan–Meier curve illustrating visual acuity stability post-BI-250 for the UG group (teal) vs. OFG group (red). Censored cases appear as a dot

**Fig. 9:**
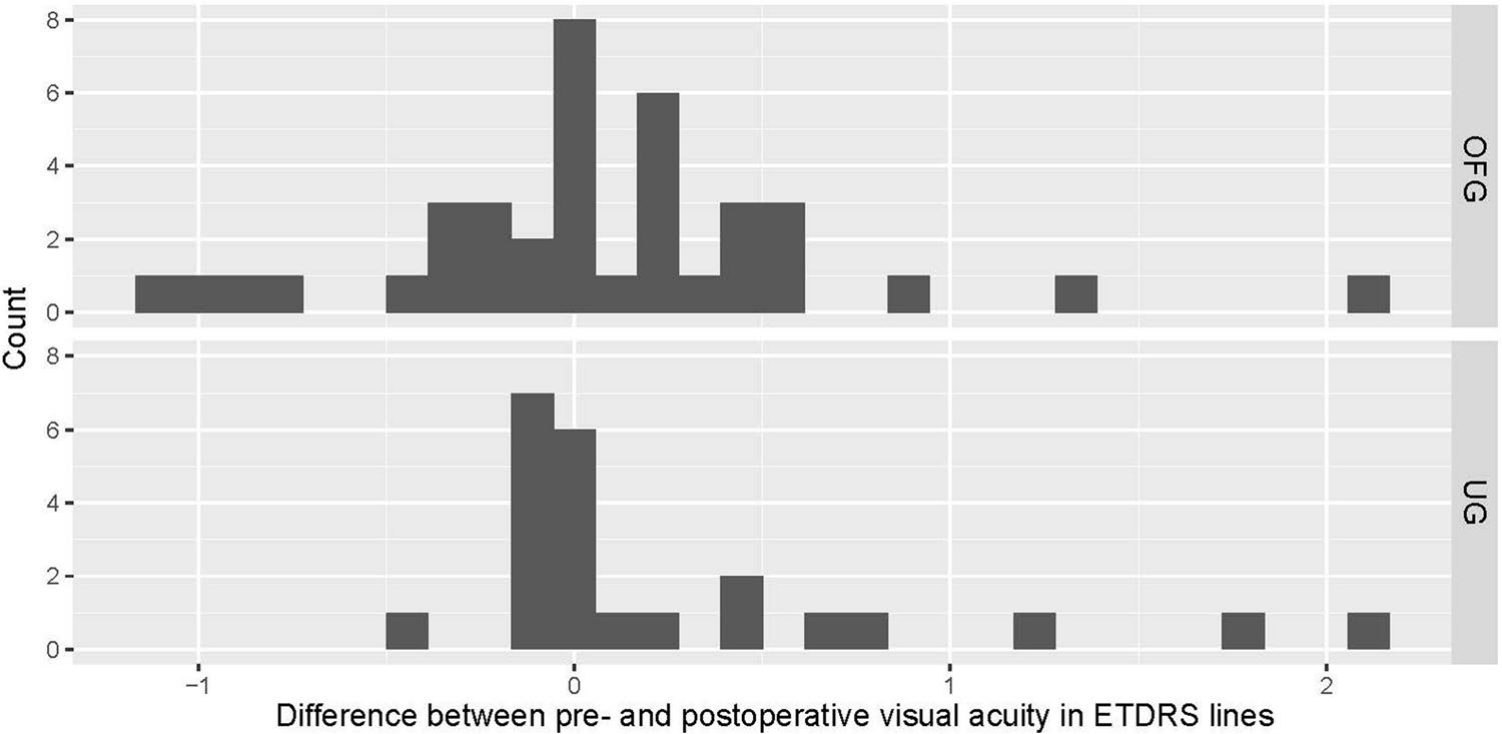
Visual acuity change post-BI-250 for the UG and OFG

Uveitic eyes experienced mostly transient visual acuity decreasing events, while the OFG group suffered a more lasting damage in part due to corneal deficits necessitating corneal transplantation (penetrating keratoplasty or Descemet membrane endothelial keratoplasty, DMEK). While neither of the two corneal transplant cases in the UG group required re-transplantation following the BI-250, 31.25% of the OFG group did require re-transplantation. Of these, 4 eyes underwent penetrating keratoplasty and 1 DME keratoplasty (Figs. 6, 7, and 8). In contrast, only 4.54% /13.63% of native corneas in either group respectively needed a keratoplasty (UG: 1 DMEK, OFG: 2 DMEKs and 1 penetrating keratoplasty). 57.89% of eyes in the OFG group maintained or improved their visual acuity by the last follow-up, as did 58.33% of eyes in the UG. No cases experienced complete loss of light perception (see Fig. 9).

Hypotony was the most common BI-250-specific complication in both groups. It affected 25% of eyes in the UG group, half of which required surgical revision. 18.42% of eyes in the OFG group experienced hypotony, which led to surgical revision in 71.43% and tube-endothelial contact in 42.86%. Additionally, diplopia incidence following implantation was comparable in both groups. Table 2 summarizes all BI-250-specific post-operative complications.

**Table 2:** Post-operative complications in both UG and OFG groups

Post-operative complications		
	UG (%)	OFG (%)
Hypotension (< 5 mmHg)	25	18.42
Revision due to hypotony	50	71.43
Endothelial-touch due to hypotony	0	42.86
Inflammation	4.17	0
Infection	0	0
Tube dislocation	0	0
Diplopia	4.17	5.26
Expulsive hemorrhage	0	2.63
Retinal detachment	4.17	0
Hypertension	0	5.26

## Discussion

Our study offers an in-depth look at post-BI-250 results stemming from complex uveitis and non-uveitis cohorts after approximately 2 years. Considering the severity of the underlying condition, we found the BI-250 to be an efficient form of treatment for both groups, relieving 75% of uveitis and 72.5% of non-uveitis eyes from further pressure decreasing surgical events.

When used as a first line of surgical treatment, the BI has yielded positive results regardless of the underlying glaucoma condition. More specifically, it has shown repeated efficacy and safety in uveitis burdened eyes [9, 15, 17, 19]. Ramdas et al. provide a comparison of GDD outcomes for eyes with vs. without secondary inflammatory glaucoma [9]. Because of similar performance results for both Ahmed and Baerveldt 350 implants, they analyzed both devices as one unit [9]. A significant and similar IOP reduction was noted in both groups, with 73.7% of uveitic and 62.3% of non-uveitic eyes reaching drug-free normotension after 1 year (uveitic glaucoma: from 25.9 ± 7.7 to 12.7 ± 4.4 mmHg (44.9% decrease; *p* < 0.001); non-uveitic glaucoma: from 27.9 ± 9.6 to 13.3 ± 4.2 mmHg (42.8% decrease; *p* < 0.001) (*p* = 0.729)) [9]. Our study expands on these insights, offering an in depth analysis of BI-250 efficacy and safety for a complex study population after almost 2 years. Since our population is different to available comparisons, juxtaposing our results is highly challenging [9, 15, 17, 19]. Our institution reserves the BI-250 as a “last resort” for refractory eyes after repeated medical and surgical treatment failure. On average, each eye in both groups had undergone one to two previous pressure decreasing interventions (UG: 1.21 (± 0.66)), OFG: 1.74 (± 1.33). In most cases of hypertension caused by uveitis, relative IOP reduction is primarily achieved through trabeculectomy ab interno [8]. Often and when necessary, this is easily combined with a simultaneous cataract extraction (pseudophakia: 79.17% UG/89.47% OFG). In our study, 42.10% of the non-uveitic eyes also underwent some form of corneal transplantation. Conjunctival scarring and scleral deterioration that result from multiple procedures, together with a decreased risk of post-operative movement restriction resulting in double vision, led to our surgeons opting for the small 250 mm^2^ BI. This contrasts with previous reports, which mostly favor the medium 350 mm^2^ BI for secondary inflammatory glaucoma [9, 15, 17, 19]. While Britt et al. conclude that there is no additional advantage to an increased filtration area [20], we cannot be certain that a larger implant would not have altered our results.

A complete success was registered by 15%/25% of eyes in the UG/OFG group, while 52.5%/32.5% of UG/OFG cases experienced a qualified success post-operatively. Furthermore, 15% of the UG and 30% of the OFG eyes no longer required topical medication, 47.5%/47.5% were free of acetazolamide systemically, and 75%/72.5% of eyes were spared from further pressure-related interventions. At first glance, our outcomes seem to be slightly inferior to other studies reporting on inflammatory glaucoma treated with a BI. When examining 24 uveitic eyes that had undergone a BI due to refractory medical treatment, Ceballos and colleagues reported a success rate of 91.7% at 24 months, with 58.3% eyes free of antiglaucoma medications at last follow-up [15]. Furthermore, 39 patients examined by Tan et al. after 24 months reached an average IOP of 10.9 ± 4.9 mmHg with 1.1 ± 1.3 medications and 44% of eyes no longer requiring any type of pressure reducing drugs [17]. Additionally, Iverson et al. found no statistical difference between 23 eyes receiving either a 350-mm^2^ (*n* = 17) or 250-mm^2^ (*n* = 6) BI, and the 5-year failure rate of the BI was 25% [19]. The meta-analysis conducted by Ramdas et al. then showed that 6–65% of eyes suffering from uveitic glaucoma no longer used any IOP-lowering medication after the implantation of either an Ahmed FP7 or a Baerveldt-350 drainage device (compared to 3–83% in the glaucoma mixture studies) [9]. This emphasizes the wide spectrum of outcomes resulting from heterogeneous study populations and consequently the necessity for supplementary data. The eyes we examined generally showed a higher starting pressure and had undergone more glaucoma- and non-glaucoma-related operations. This can in part be attributed to lengthy preexisting underlying uveitic conditions (averaging 146.72 months). The severity of the pre-surgical ocular status of both groups can be deduced from the uncontrolled elevated pressure that persisted despite over 75% of eyes receiving systemic carbonic anhydrase inhibitors, which are more potent than their topical counterparts [21]. As previously mentioned, the relevance of the BI size can also not be excluded from the interpretation of our data as no previous studies have primarily reported on the 250 mm^2^ BI.

Regarding sight-threatening events, our UG group experienced more cases of decreased visual acuity post-operatively due to macular edema, while the OFG group’s visual acuity drop was mostly caused by corneal edema. This is concordant with meta-analyses that show post-operative macular edema development in 2–26% of inflammatory glaucoma vs. 2–4% in glaucoma mixture studies [9]. Our 40% prevalence is higher than previous accounts. In a study with over 70% anterior uveitis, Iverson et al. report macular edema developing in 26% of eyes following Baerveldt implantation [19]. Macular edema is less common in anterior uveitis compared to eyes suffering from intermediate-, posterior-, or pan-uveitis [22–24]. Therefore, we can attribute our increased occurrence to the inclusion of 46% intermediate- or pan-uveitis. To date, no studies have thoroughly examined the prevalence of corneal edema post-BI-250 in uveitic glaucoma. Available mixture glaucoma reports show a rate of persistent corneal edema ranging from 5.6 to 17.5% [25, 26]. Corneal endothelium deteriorates at a rate of 0.6% yearly [27]. After undergoing trabeculectomy ab externo, the rate increases to about 10% per year [28, 29]. In contrast, no significant change is experienced after trabectome surgeries [30]. While the majority of uveitic eyes in our study had previously undergone a trabeculectomy ab interno, many of the OFG group underwent an ab externo trabeculectomy with MMC. This could partially explain the slightly higher initial corneal edema incidence in the OFG group. Additionally, 42.10% of these eyes had received some form of keratoplasty prior to the BI-250. Considering these factors, as well as the accelerated endothelial cell loss experienced after corneal transplantation [31], corneal edema susceptibility could also be due to the presence of a corneal graft. Furthermore, 31.25% of grafts necessitated a re-transplantation after BI-250 surgery, while only 13.63% of native corneas required a keratoplasty. 57.89% of eyes in the OFG group and 58.33% of eyes in the UG group either maintained or improved their visual acuity post-operatively. Tan et al. report a clinically relevant visual acuity loss in 34% of uveitic eyes [17]. However, one cannot directly juxtapose results since baseline visual acuities are significantly different between both studies.

Hypotony manifests in 1–38% of eyes in heterogeneous glaucoma cohorts and in 11–36% of secondary inflammatory glaucoma post-BI [9]. In our study, 25% of UG vs. 18.42% of OFG eyes experienced transient hypotony, which was resolved in 50% and 71.43% of cases respectively through surgical revision. The decreased pressure phase led to endothelial contact in 0% vs. 42.86% of UG/OFG eyes. Additionally, 5.26% of eyes in the OFG group required surgical/laser interventions due to persistent hypertension caused by a fibrotic membrane and an unabsorbed tube suture. Available data show revision rates ranging from 6 to 13% in mixed glaucoma eyes and 0% in uveitic eyes [9]. Worth noting is the fact that only 4 of the 15 studies included in Ramdas et al.’s meta-analysis reported on secondary surgeries related to BIs. Moreover, the presence of mild diplopia in 4.17% of our UG group using the BI-250 is significantly below the 15% reported by Tan et al., who mainly implanted the larger 350 mm^2^ device [17].

## Conclusion

Our data shows that the BI-250 is an effective and safe intervention for advanced glaucoma cases in uveitis as well as other forms. The majority in both our groups required no further glaucoma-related surgery within the follow-up period of almost 2 years and either maintained or improved visual acuity by the last examination. Nevertheless, sight-threatening complications including macular and corneal edema occur frequently, and grafted eyes are prone to corneal decompensation, which can lead to re-transplantation.

The advent of biologicals has provided a means of successfully controlling uveitis and its underlying condition, thus delaying the structural alterations that lead to increased ocular pressure. Moreover, the myriad of conservative alternatives and microinvasive procedures can further postpone the necessity of a highly invasive intervention. Since the BI-250 has shown high efficacy and safety in both native and surgically burdened eyes, the relevant question to be asked in further studies is not if, but when is the correct time to act drastically?
